# The Validity and Reliability of a Smartphone Application for Measuring Wrist and Metacarpophalangeal Joint Motion

**DOI:** 10.7759/cureus.58047

**Published:** 2024-04-11

**Authors:** Ömer Faruk Özçelep, Melek Güneş Yavuzer, Ayşe Nur Tunali

**Affiliations:** 1 School of Physical Therapy and Rehabilitation, Ahi Evran University, Kırşehir, TUR; 2 Department of Health Sciences, Haliç University, Istanbul, TUR; 3 Department of Physiotherapy and Rehabilitation, Istanbul Medipol University, Istanbul, TUR

**Keywords:** range of motion (rom), wrist evaluation, metacarpophalangeal joint, mobile phone application, reliability and validity

## Abstract

Background

Accurate measurement of the range of motion (ROM) is crucial for guiding upper extremity rehabilitation. Inaccurate measurements can mislead clinicians and harm patient compliance. This study aimed to evaluate the validity and reliability of a smartphone application (Angulus) for measuring wrist and metacarpophalangeal (MCP) joint ROM.

Methodology

This study included 64 volunteers with no prior wrist injuries. The wrist flexion/extension, radial/ulnar deviation, and MCP joint flexion/extension ROM were assessed by an experienced physiotherapist (Assessor 1) using the Angulus and a standard two-arm goniometer. The validity of Angulus was evaluated by correlating it with the goniometer measurements using the Pearson correlation coefficient. For the reliability analysis, an inexperienced biomedical engineer (Assessor 2) performed the same measurements using Angulus twice in different sessions, in addition to Assessor 1. The intra-rater and inter-rater reliability were tested using the intraclass correlation coefficient.

Results

The mean age of the participants was 29.5 ± 8.2 years, with 62% being female. The validity of the Angulus app measurements was indicated by the adequate to excellent correlation between the Angulus and goniometer measurements (ranging from 0.44 to 0.81). The intra-rater reliability of the Angulus app was excellent for Assessor 1 (ranging from 0.76 to 0.90) and adequate to excellent for Assessor 2 (ranging from 0.71 to 0.88). The inter-rater reliability of Angulus was excellent (ranging from 0.91 to 0.96).

Conclusions

Angulus is a valid and reliable method to measure the wrist and MCP joint ROM.

## Introduction

The hand and wrist are the most active parts of the upper extremity and are more susceptible to damage that can cause dysfunction [1]. Muscle weakness, deformity, edema, and pain can lead to a loss of range of motion (ROM), resulting in reduced fine manipulation, grasping ability, and grip strength [2]. Wrist and hand examinations require careful investigation to avoid incorrect diagnosis, which can lead to ineffective treatment [3]. It may lead to delayed return to work, reduced performance and function, pain, degenerative joint disease, and increased medical costs [4]. Wrist ROM is a critical measure in the clinical setting, essential for diagnosis, treatment evaluation, and quantification of potential changes [5]. In clinical practice, goniometers are presently the gold standard [6,7]. Wrist ROM measurements are obtained using a manual goniometer, a non-invasive and low-cost instrument [8]. However, ROM measurements require an experienced clinician for reliable results, which increases the costs for both patients and clinicians [9].

Smartphones with high-resolution photos are a potential platform for patient ROM assessment [9,10]. Recently, several smartphone applications have become available that provide an alternative method of monitoring joint angles [11]. Wassmuth et al. found the Goniometer PRO to be an effective tool for evaluating wrist ROM [12]. Similar results were found in forearm supination using the DrGoniometer, which is a valid and alternative tool for measuring ROM [13]. It is quick and easy to take a picture, which is an advantage for irritable patients [14]. The digital record created is useful for comparison during follow-up treatments, telerehabilitation, and medical purposes [15]. According to Alford, a person’s ability to self-monitor ROM gains at home can lead to increased motivation and compliance with home exercise programs [7]. This study aimed to determine if the Angulus application is a valid and reliable method for measuring wrist and metacarpophalangeal (MCP) joint ROM without the need for clinical experience.

This article was previously presented as a meeting abstract at the 2023 Federation of European Societies for Surgery of the Hand Congress on May 10-13, 2023.

## Materials and methods

Participants

A total of 64 volunteers between the ages of 18-50 years who signed an informed consent form were enrolled in the study at Istanbul Medipol University Çamlıca Hospital. Exclusion criteria were the presence of pain in the hand and wrist and the diagnosis of orthopedic, neurologic, and rheumatologic diseases. The study protocols were approved by the Haliç University Non-interventional Clinical Research Ethics Committee (approval number: 27.05.2021/110).

Evaluating procedures

**Figure 1:**
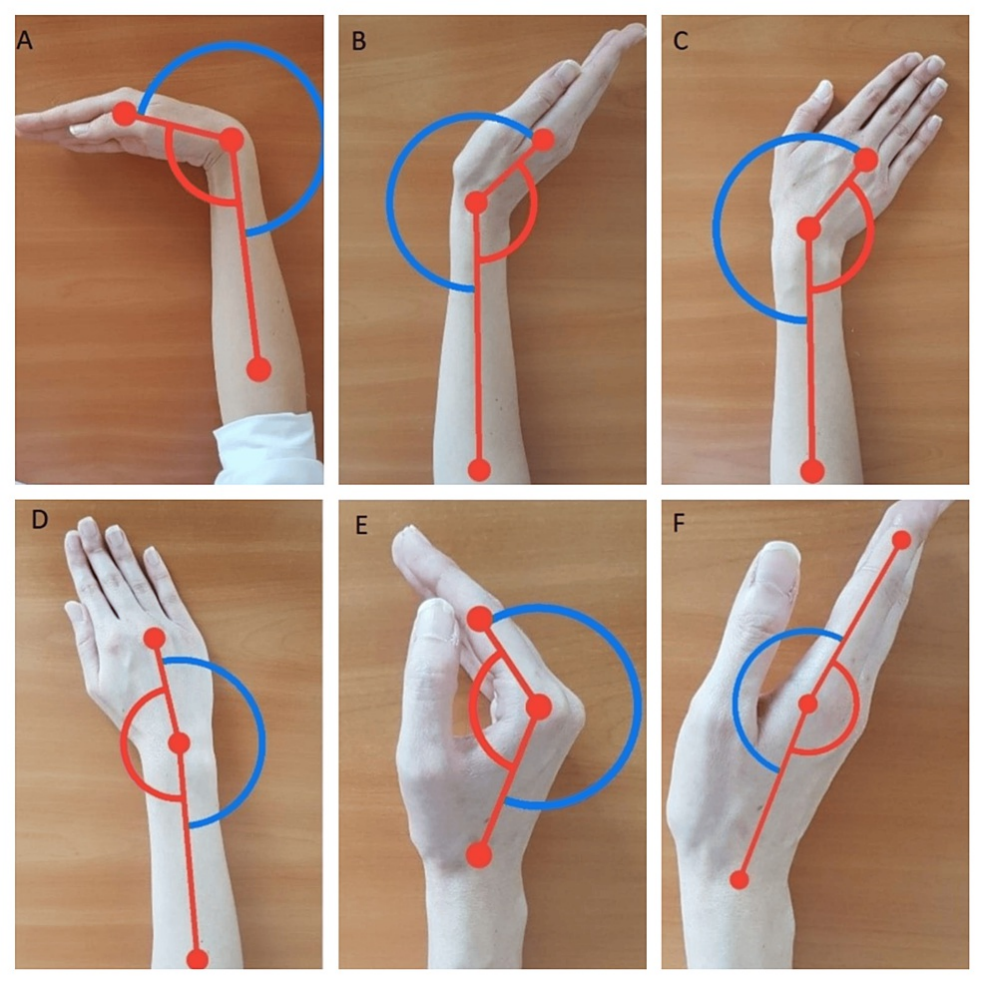
Angulus measurements. (A) Wrist flexion. (B) Wrist extension. (C) Ulnar deviation. (D) Radial deviation. (E) Metacarpophalangeal flexion. (F) Metacarpophalangeal extension.

**Figure 2:**
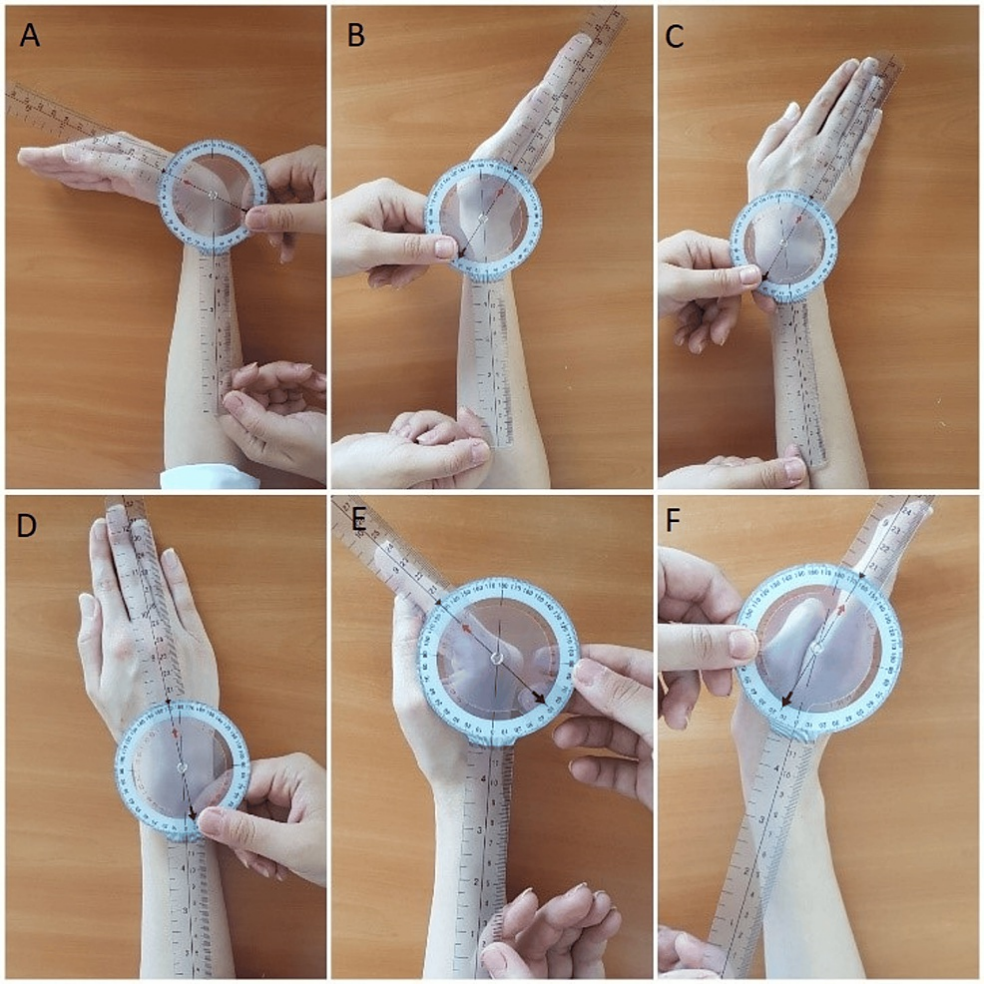
Goniometric measurements. (A) Wrist flexion. (B) Wrist extension. (C) Ulnar deviation. (D) Radial deviation. (E) Metacarpophalangeal flexion. (F) Metacarpophalangeal extension.

Angulus (DPP, USA) is an application for mobile phones that measures joint angles from photographs or videos captured by the phone’s built-in camera. The software allows the user to measure the joint angle by drawing lines and points over the image. Basic photography skills are required to use the application, and the instructions for the user of the various wrist and MCP joint movements are straightforward. Therefore, the application is suitable for clinicians and researchers studying wrist pathology. Two assessors, a physical therapist (Assessor 1) and a biomedical engineer (Assessor 2), performed the measurements on the subject’s dominant side. The goniometer measurements were followed by Angulus measurements, and this order was maintained for measurements performed at different times. Assessor 1 has worked in the field for six years and specializes in hand rehabilitation, while Assessor 2 was unfamiliar with ROM measurements. The assessors performed the measurements individually. Subjects were comfortably seated in a chair with their hands on the table, shoulders slightly abducted, and elbows at 90 degrees. Wrist flexion/extension and MCP joint flexion/extension ROMs were measured in the neutral position, while ulnar/radial deviation was measured in the pronated position. To assess validity, Assessor 1 used the Angulus application (Figure 1) and a standard two-arm goniometer (Figure 2) to measure the patients’ wrist flexion/extension, radial/ulnar deviation, and MCP flexion/extension ROMs.

Goniometric measurements were performed three times and the higher value was recorded. After the goniometric assessments, a 10-minute break was taken to prepare for the Angulus app measurements. Photographs of the same hand positions were taken at a distance of 60 cm, perpendicular to the MCP joint, with smartphone cameras (Samsung S8 and J7-Core), separately by two assessors, twice, with a five-minute break in between. At the end of the day, Assessor 1 and Assessor 2 separately performed the Angulus app measurements using photographs and entered the measurement data into their participant evaluation forms. The entire measurement process took approximately 45 minutes for each participant.

Statistical analyses were performed using SPSS version 24 (IBM Corp., Armonk, NY, USA). Mean values and standard deviations (SDs) were used for descriptive purposes [16]. Shapiro-Wilk test was used to establish the normality of the data, and the statistically significant level was determined as p-values <0.05. Due to the normal distribution of the data, the validity of the Angulus app measurements was assessed by Pearson correlation coefficient (r). The Pearson correlation coefficient values were classified as <0.30, 0.30-60, and >0.60 being poor, adequate, and excellent, respectively. Intraclass correlations (ICC 2.1, two-way random effects absolute agreement) with 95% confidence intervals (CIs) were used to measure the inter- and intra-rater reliability for the joint ROM [17]. ICC values were classified as poor (<0.40), adequate (0.40-0.75), and excellent (>0.75) [18].

## Results

All 64 volunteers (40 females, 59 right-handed) completed the study without adverse events. The mean age was 29.5 ± 8.2 years. The demographic characteristics of the participants are shown in Table 1.

**Table 1:** Demographic characteristics of the participants. Min = minimum; Max = maximum; SD = standard deviation

	Min–Max	Mean ± SD
Age	20–48	29.5 ± 8.21
Height	1.55–1.88	1.68 ± 0.08
Weight	43–102	71 ± 15.8
Body mass index (kg/m^2^)	17–39	25 ± 4.45

The measurements of Assessor 1 using the Angulus app and a standard goniometer for validity assessment are shown in Table 2.

**Table 2:** Measurements of Assessor 1 performed by the Angulus app and standard goniometer for validity assessment. r = Pearson correlation coefficient; Min = minimum; Max = maximum; SD = standard deviation; G = gonimeter; A = Angulus; MCP = metacarpophalangeal

Assessor 1	Min–Max	X ± SD	r
Wrist flexion (G)	50–94	69.3 ± 9.58	0.791
Wrist flexion (A)	40–93	64.9 ± 9.95	
Wrist extension (G)	36–80	61.7 ± 8.09	0.537
Wrist extension (A)	28–77	58.4 ± 9.54	
Wrist ulnar deviation (G)	27–72	51 ± 8.65	0.675
Wrist ulnar deviation (A)	26–61	43.3 ± 7.77	
Wrist radial deviation (G)	0–40	23.7 ± 7.49	0.716
Wrist radial deviation (A)	0–46	19.4 ± 8.15	
MCP joint flexion (G)	65–109	81.3 ± 9.44	0.434
MCP joint flexion (A)	44–86	68.9 ± 9.17	
MCP joint extension (G)	0–50	24.4 ± 11.46	0.814
MCP joint extension (A)	0–47	27.8 ± 12.17	

The correlation between the two methods was excellent for wrist flexion (r = 0.80), radial deviation (r = 0.71), ulnar deviation (r = 0.68), and MCP joint extension (r = 0.81) and adequate for wrist extension (r = 0.54) and MCP joint flexion (r = 0.44). The first and second measurements of Assessor 1 and Assessor 2 for intra-rater reliability of the Angulus application are shown in Table 3.

**Table 3:** First and second measurements of Assessor 1 and Assessor 2 for intra-rater reliability of the Angulus app. Min = minimum; Max = maximum; SD = standard deviation; MCP = metacarpophalangeal; ICC = intraclass correlation

	Angulus app	Min–Max	Mean ± SD	ICC
Assessor 1	Wrist flexion 1st	40–93	64.9 ± 9.95	0.801
	Wrist flexion 2nd	40–88	64.7 ± 9.28	
	Wrist extension 1st	28–77	58.4 ± 9.54	0.797
	Wrist extension 2nd	35–77	57.6 ± 9.90	
	Wrist ulnar deviation 1st	26–61	43.3 ± 7.77	0.795
	Wrist ulnar deviation 2nd	26–64	43.4 ± 7.90	
	Wrist radial deviation 1st	0–46	19.4 ± 8.15	0.820
	Wrist radial deviation 2nd	0–47	19.5 ± 8.82	
	MCP joint flexion 1st	44–86	68.9 ± 9.17	0.764
	MCP joint flexion 2nd	53–85	68.3 ± 7.76	
	MCP joint extension 1st	0–47	27.8 ± 12.17	0.901
	MCP joint extension 2nd	0–46	28.4 ± 12.30	
Assessor 2	Wrist flexion 1st	41–88	66.5 ± 11.25	0.873
	Wrist flexion 2nd	42–87	65.1 ± 11.63	
	Wrist extension 1st	33–69	53.3 ± 8.13	0.882
	Wrist extension 2nd	35–70	53 ± 8.50	
	Wrist ulnar deviation 1st	24–62	41.9 ± 7.47	0.714
	Wrist ulnar deviation 2nd	16–57	41.5 ± 8.25	
	Wrist radial deviation 1st	0–51	18.5 ± 8.46	0.877
	Wrist radial deviation 2nd	0–45	18.2 ± 7.71	
	MCP joint flexion 1st	56–95	78 ± 7.26	0.811
	MCP joint flexion 2nd	54–96	77 ± 8.27	
	MCP joint extension 1st	0–47	23.8 ± 10.98	0.827
	MCP joint extension 2nd	0–76	24.8 ± 12.80	

The ICCs for intra-rater reliability of the Angulus app were excellent (ranging from 0.76 to 0.90) for all measurements for both assessors, except for the wrist ulnar deviation of Assessor 2 (0.71), which was adequate. ICCs for inter-rater reliability of the Angulus app were excellent (ranging from 0.91 to 0.96) (Table 4).

**Table 4:** Measurements of Assessor 1 and Assessor 2 for inter-rater reliability of the Angulus app. Min = minimum; Max = maximum; SD = standard deviation; MCP = metacarpophalangeal; ICC = intraclass correlation

Angulus app	Min–Max	Mean ± SD	ICC
Wrist flexion (Assessor 1)	40–93	64.9 ± 9.95	0.933
Wrist flexion (Assessor 2)	41–88	66.5 ± 11.25	
Wrist extension (Assessor 1)	28–77	58.4 ± 9.54	0.944
Wrist extension (Assessor 2)	33–69	53.3 ± 8.13	
Wrist ulnar deviation (Assessor 1)	26–61	43.3 ± 7.77	0.910
Wrist ulnar deviation (Assessor 2)	24–62	41.9 ± 7.47	
Wrist radial deviation (Assessor 1)	0–46	19.4 ± 8.15	0.950
Wrist radial deviation (Assessor 2)	0–51	18.5 ± 8.46	
MCP joint flexion (Assessor 1)	44–86	68.9 ± 9.17	0.908
MCP joint flexion (Assessor 2)	56–95	78 ± 7.26	
MCP joint extension (Assessor 1)	0–47	27.8 ± 12.17	0.960
MCP joint extension (Assessor 2)	0–47	23.8 ± 10.98	

## Discussion

Recent studies have reported the validity and reliability of various smartphone applications for measuring hand wrist ROM. Two types of apps can be distinguished, i.e., apps that use the smartphone’s built-in accelerometer and apps that work with images, called photogoniometers. No significant differences in reliability and validity were found between these two groups [19]. According to Valdes et al., mobile device applications can be a valuable intervention and impact performance in individuals with impaired hand function. Applications provide a client-centered and potentially motivating activity option that can be used to assist the hand therapist [20]. In addition, Mitchell et al. assessed the reliability and validity of accelerometer-based and photo-based goniometric applications and suggested that photo-based applications may offer a more appropriate measurement method by providing a permanent, printable protocol [21]. The application used in our study offers the possibility of evaluation through photography. This feature distinguishes it from accelerometer-based applications. According to Ferrierio et al. [15], photo-based applications have several advantages: clinicians can measure the photo at any time; they can be used in telemedicine to monitor patients at home; and they can help improve patient compliance by showing the patient consecutive images that reveal the change. Because it does not require contact with the skin, photo-based measurement can be used in the operating room without special preparation. Cunha et al. reported that video-based goniometry could be used as a new standard goniometric technique because it is clinically validated and performed with greater consistency and excellent inter-rater reliability compared to goniometry. It also allows for better documentation of measurements and potential direct incorporation into medical records [22]. Ge et al. found smartphone photographs to be a valid and reliable method of measuring wrist ROM and an alternative to manual measurements using goniometers to assess functional outcomes of the wrist. According to Reid et al., DrGoniometer is an equivalent tool for measuring forearm supination compared to universal goniometer in healthy populations and populations with known forearm fractures [9,13]. In this study, we found similar results to the above-mentioned studies because the inter-rater and intra-rater validity and reliability were considerably higher in our study compared to the manual goniometer.

In all of the above-mentioned studies, assessors were selected from clinicians experienced in hand evaluation. Hand surgeons performed the assessments, or they were performed by researchers or raters whose expertise we could not obtain. Contrary to these studies, one of the evaluators in our study was an expert physiotherapist, while the other was a biomedical engineer with no experience in hand assessment. In our study, regardless of experience, inter-rater reliability was above 0.90, similar to the study of Mitchell et al. in shoulder external rotation. In the above-mentioned studies, except for Trehan et al., the photographs were assessed with computer programs such as Image J and Adome CS5. Trehan et al. evaluated photographs by placing a goniometer. In our study, the angle between the lines that appear when the evaluator determines the points on the photograph was calculated automatically. We believe this was the main difference that made the assessment faster than the computer [21,23].

DrGonimeter showed excellent intra-rater reliability and good validity. However, inter-rater reliability was good to excellent in the fractured forearm group but poor to fair in the normal population. In our study, the correlation between goniometric ROM measurements and Angulus app was above 0.70, except for wrist extension (0.54) and MCP flexion (0.44), indicating adequate to excellent validity. In addition, ICCs were above 0.75, except for wrist ulnar deviation of Assessor 2 (0.71), indicating adequate and excellent reliability.

To minimize errors, it is necessary to strictly follow the correct procedures described in the validation studies for all measurement methods. In particular, the photo-based applications are subject to the same limitations as standard digital photographic goniometric measurements and have two main potential sources of error: (i) unstable handling of the smartphone when taking the picture, and (ii) imprecision in positioning the virtual goniometer on the mobile screen. The app is also convenient for use in clinical trials, as the photograph and data generated can be consulted if a measurement error is suspected, as was found in our study. Another advantage of the Angulus app is that once the app’s dots are placed on the photograph, the app automatically calculates and displays the angle to reduce calculation errors that can occur when reading the measurement from a universal goniometer. The entire measurement process is slower when using the Angulus app to measure joint angles than when using a universal goniometer. Although the snapshot can be taken quickly to reduce the time the patient must remain in an uncomfortable position, the user must then save the image and apply the app’s measurement points. Aligning the arms and reading the measurement directly from the universal goniometer can take longer. As a result, the use of the Angulus app may be impractical in some time-constrained situations, which may discourage clinicians from using the app. Future studies in this area may be qualitative to determine the challenges of using the Angulus app.

Limitations

The robust study design according to the guidelines for reporting reliability and concordance studies and the sufficient number of subjects to assess the correlation between intra- and inter-rater reliability with statistically significant results are the main strengths of our study. The first of the main limitations was that as ROM was measured by two evaluators at different settings, the wrist had to be perfectly positioned to have comparable angles during manual measurements and photographic measurements. We placed the smartphone’s camera at a certain distance from the exact line of the evaluated joint. However, this placement varies according to the person’s photographing skills and the patient’s compliance. Another major limitation was the lack of population diversity. Because this study was designed for healthy participants, the results will not be accurate for any disease population. In addition, it is one of the disadvantages of this application that the points in the photographs are difficult to match with the anatomical signs. Experienced clinicians will place the points in the anatomically correct places on the application, while inexperienced clinicians will have a hard time placing the points. Therefore, for a reliable assessment, a guide should show users how to take pictures.

## Conclusions

This study was designed to determine if the Angulus application is a valid and reliable method of measuring wrist and MCP ROM without the need for clinical experience. This study demonstrated that the Angulus application is a reliable and valid alternative to wrist and MCP goniometry. It was also concluded that lack of clinical experience was not a barrier to using the app and therefore could be incorporated into telerehabilitation practices. Further research may show that these tools can be effective as an adjunct to clinical practice or as part of a home program for therapists or patients who prefer to measure wrist and hand ROM using a smartphone-based app.
